# Supplementary material for: Resolving the full spectrum of human genome variation using Linked-Reads
Source: Genome Res. 2019 Apr;29(4):635–45. doi: 10.1101/gr.234443.118 (PMC6442396; doi:10.1101/gr.234443.118)
Supplement: Supplemental Material [file supp_gr.234443.118_Supplemental_longranger-2.2.zip › longranger-2.2.tar.bz2!/lr2.2-public/lib/stan/cmdstan/stan/doc/api/html/mcmc_8hpp_source.html]

Stan: src/stan/mcmc.hpp Source File


|  |  |
| --- | --- |
|  | Stan  2.5.0  probability, sampling & optimization |


- Main Page
- Related Pages
- Namespaces
- Classes
- Files

- File List
- File Members

All Classes Namespaces Files Functions Variables Typedefs Enumerations Enumerator Friends Macros Pages

- src
- stan

mcmc.hpp

Go to the documentation of this file.

1 #ifndef STAN\_MCMC\_HPP

2 #define STAN\_MCMC\_HPP

3

4 #include "stan/mcmc/chains.hpp"

5 #include "stan/mcmc/sample.hpp"

6

7 #include "stan/mcmc/hmc/static/unit\_e\_static\_hmc.hpp"

8 #include "stan/mcmc/hmc/static/diag\_e\_static\_hmc.hpp"

9 #include "stan/mcmc/hmc/static/dense\_e\_static\_hmc.hpp"

10 #include "stan/mcmc/hmc/static/adapt\_unit\_e\_static\_hmc.hpp"

11 #include "stan/mcmc/hmc/static/adapt\_diag\_e\_static\_hmc.hpp"

12 #include "stan/mcmc/hmc/static/adapt\_dense\_e\_static\_hmc.hpp"

13

14 #include "stan/mcmc/hmc/nuts/unit\_e\_nuts.hpp"

15 #include "stan/mcmc/hmc/nuts/diag\_e\_nuts.hpp"

16 #include "stan/mcmc/hmc/nuts/dense\_e\_nuts.hpp"

17 #include "stan/mcmc/hmc/nuts/adapt\_unit\_e\_nuts.hpp"

18 #include "stan/mcmc/hmc/nuts/adapt\_diag\_e\_nuts.hpp"

19 #include "stan/mcmc/hmc/nuts/adapt\_dense\_e\_nuts.hpp"

20

21 #endif

unit\_e\_static\_hmc.hpp

chains.hpp

unit\_e\_nuts.hpp

adapt\_unit\_e\_nuts.hpp

adapt\_unit\_e\_static\_hmc.hpp

adapt\_dense\_e\_static\_hmc.hpp

dense\_e\_static\_hmc.hpp

dense\_e\_nuts.hpp

adapt\_dense\_e\_nuts.hpp

diag\_e\_static\_hmc.hpp

diag\_e\_nuts.hpp

sample.hpp

adapt\_diag\_e\_static\_hmc.hpp

adapt\_diag\_e\_nuts.hpp


---

[ Stan Home Page ]

*© 2011–2014,
Stan Development Team.*
